# A qualitative Exploration of Contextual Factors Within Schools Impacting the Introduction of the New Statutory ‘Framework on Embedding a Whole School Approach to Emotional and Mental Wellbeing’ in Wales

**DOI:** 10.1007/s12310-024-09740-z

**Published:** 2025-01-08

**Authors:** Rachel Brown, Rebecca Anthony, Olga Eyre, Jessica Lennon, Vicky Powell, Zoe Haslam, Abbey Rowe, Graham Moore

**Affiliations:** 1 Wolfson Centre for Young People’s Mental Health, Spark, Cardiff University, Maindy Road, Cardiff, CF24 4HQ UK; 2 DECIPHer, Spark, Cardiff University, Maindy Road, Cardiff, CF24 4HQ UK

**Keywords:** Whole school approach, Policy, Implementation, Mental health, Emotional wellbeing

## Abstract

In 2021, the Welsh Government introduced new statutory guidance for schools titled ‘Framework Guidance on Embedding a Whole School Approach to Emotional and Mental Wellbeing’. This document outlined new responsibilities for educational settings to work towards incorporating a whole school approach, with regard to the Framework in action planning, service delivery and policy in relation to the mental and emotional wellbeing of learners and staff. While there is growing evidence to suggest that whole school approaches can be beneficial to social and emotional wellbeing for pupils, evidence on effective implementation is limited. This paper reports on findings from qualitative group interviews with staff in schools in Wales, conducted as part of a wider, mixed-methods evaluation of the Framework. It focuses on factors in the school context which impacted initial implementation. A number of school level factors were identified as barriers to implementation and staff engagement with the Framework. School staff reported higher levels of pupil mental health challenges stemming from the COVID-19 pandemic and an absence of capacity in in-house and external support services to address this. Poor staff wellbeing and significant workload pressures were also reported, driven in part by concurrent implementation of the new Curriculum For Wales. This led staff to feel ill-prepared for the more complex issues being faced, exacerbated by a lack of access to relevant training to be able to support pupil and colleague mental health. This paper concludes with recommendations for policy-makers to support Framework implementation.

## Introduction

Evidence suggests a deterioration in mental health among both the youth and adult population of the UK in recent years (Hu & Qian, 2021; Pierce et al., 2020). In response, there has been increasing emphasis in policy on the role of schools as a setting for mental health improvement for young people, reflecting known benefits of strong relationships with school staff and a sense of belonging on pupil mental health (Long et al., 2021).

There is growing evidence to suggest that whole school approaches can be beneficial to social and emotional wellbeing for pupils (Bonell et al., 2018; Goldberg et al., 2019). A whole school approach is a systemic intervention aimed at change across multiple system levels and including input from all stakeholders in and around the school setting (Quinlan & Hone, 2020). In March 2021, the Welsh Government introduced new statutory guidance for educational settings in Wales, titled ‘Framework Guidance on Embedding a Whole School Approach to Emotional and Mental Wellbeing’ (hereafter referred to as ‘the Framework’) (Welsh Government, 2021). The Framework outlined a programme of work to promote a positive school culture supportive of mental wellbeing, with new responsibilities for all educational settings to plan, deliver and create policies to incorporate a whole school approach to improve the mental and emotional wellbeing of learners and all staff. It is stated that this should be collaborative and inclusive of all key stakeholders, including families and others external to schools who are connected to their function, such as local authorities, local health services and specialist services supporting mental wellbeing (Welsh Government, 2021).

Evidence on effective implementation and long-term monitoring of outcomes for whole school approaches to mental health is limited (Brown et al., 2022), but suggests that identifying factors that impact initial programme implementation is key in understanding and interpreting outcomes (Durlak & DuPre, 2008; Durlak, 2016). Schools can be characterised as complex systems (Hawkins and James, 2018; Moore et al., 2019), incorporating multiple levels, being interactive and containing a diverse range of actors who behave in both autonomous and rule-driven ways (Keshavarz et al., 2020). Whether programmes become embedded within complex systems, and the effects they then give rise to, are highly contingent on place and time (Pawson & Tilley 1997), meaning issues impacting school life at any given time are likely to influence how a programme operates. Evidence suggests a range of factors within schools that are likely to impact implementation of new wellbeing programmes, including staff retention and workload and perceived fit with existing needs (Darlington et al., 2017), which impacts willingness to engage with any new activity. Schools frequently engage in practices relating to mental wellbeing which may be impacted or displaced by new initiatives, meaning that identifying existing provision is key to understanding the fit of any new initiatives (Hawe et al., 2009). Further, provision of funding and policy-level support provided to schools may be significant to both initial implementation and longer-term sustainability (Durlak & DuPre, 2008).

In an evaluability assessment of whole school approaches to mental health, pupil socio-demographic factors, staff skills and capacity, the availability of specialist mental and emotional wellbeing services, local authority roles and existing practices in relation to emotional and mental wellbeing, were all identified as likely to be of relevance in understanding programme implementation (Brown et al., 2022). This suggests that exploring the context within schools when a new programme is launched is key to understanding how it may operate.

The Framework was launched in March 2021, at a time of significant challenge, with intermittent school closures due to COVID-19 meaning in-person teaching for a limited number of children, online provision for all other pupils and a phased return for all age groups over the course of that calendar year. Other significant education system reforms also occurred around this time, including the introduction of the Additional Learning Needs (ALN) Code for Wales in March 2021, which outlined a process of complete transformation of the ALN system in education settings, as well as the impending introduction of the new Curriculum for Wales. This stemmed from the Donaldson Report (2015) and included significant changes to the provision of health and wellbeing education (https://hwb.gov.wales/curriculum-for-wales/health-and-well-being/), with plans for phased rollout in schools from September 2021. The education system at the time was therefore in flux, potentially creating challenges for implementation.

This paper reports on findings from the first round of interviews in schools in Wales (repeated a year later and to be reported separately), conducted as part of a wider, mixed-methods evaluation of the Framework (https://osf.io/5zcka). Findings here enhance understanding of barriers and facilitators to the introduction of the Framework, from the perspectives of those delivering it, and will aid policy decisions on supporting effective implementation and sustainability of the policy going forward. Staff perspectives of key contextual factors within schools at the time of implementation of the Framework are reported, relating to the following research questions:

1. What are the barriers and facilitators to changes in school practice and functioning in response to the Framework?
2. How do stakeholders working within schools respond to Framework implementation?

## Methods

This is a qualitative study involving semi-structured group interviews, conducted with school staff as part of longitudinal case studies at 12 school sites in Wales.

### Sampling and Recruitment

Case study sites were situated across Wales, in rural and urban areas and in areas with a wide range of socio-economic status (based on rates of pupils eligible for free school meals), selected to give a range of experiences of Framework implementation. Sampling for the wider case studies adopted an instrumental case study approach (Stake, 1995) whereby cases are not included as ‘typical’ or representative examples but for the opportunity they present for learning. This reflects an understanding that all complex social settings, such as schools, are unique examples of interactions between agents and processes (Stake, 2000). Hence, sampling aimed to achieve diversity, not representativeness.

Four primary (pupil age 5–11) and 7 secondary (pupil age 11–16) schools were identified, as well as one ‘through school’ which provides continuous education from age 3–18. Two of the participating schools operate in the medium of Welsh, with interviews conducted in Welsh. At all other sites, interviews took place in English. Interviewees were identified from discussions with school senior leaders. Interviewee roles varied by school but all were identified as providing pastoral support either as their primary role or as an addition to a teaching role. Job titles included: deputy heads, heads of year, heads of wellbeing, pastoral leads, additional learning needs co-ordinators and teaching assistants.

### Data Collection and Analysis

Table 1 presents outline information on the type of school and participants within each group interview. We interviewed 54 staff in total and all interviews took place within one academic year, between December 2022 and June 2023.

**Table 1:** Summary of participants

School identifier	School type	Number of interviewees
School 1	Welsh Medium All-age (3–18) School	Group interview–4 Individual interview–1
School 2	Primary	6
School 3	Secondary	4
School 4	Secondary	5
School 5	Welsh Medium Secondary	Group interview–5 Individual interview–1
School 6	All-age (3–18) school	5
School 7	Secondary	4
School 8	Primary	2
School 9	Primary	4
School 10	Secondary	6
School 11	Secondary	5
School 12	Primary	2

A semi-structured topic guide was developed for these interviews, focused on: targeted and universal provision to support emotional and mental wellbeing; contextual factors in schools at and around the time of the Framework launch; staff wellbeing; and staff understanding of—and responses to—Framework publication.

A favourable ethical opinion was provided following review by Cardiff University School of Social Sciences Research Ethics Committee. All participants were supplied with a bilingual (Welsh/English) information sheet in advance of interviews, providing details of recording, data use and storage, rights of withdrawal and intended data use, as well as researcher contact details for any queries. Participants were asked to read and complete a consent form on the day of visit. Interviews were audio recorded for later transcription by an external company and the resulting transcripts were quality checked by study research assistants, before anonymisation. Transcripts were then analysed using a reflexive thematic analysis approach (Braun & Clarke, 2019), led by the lead author and supported by a study research assistant. This involved engaging openly and reflexively with the data to develop an initial coding frame, guided by the study research questions and notable areas of interest in the data. The coding frame was applied separately to a sample of transcripts by each researcher, followed by meetings to discuss any divergence of views with review of the relevant section of the transcript. These led to minor revisions and additions to the frame, before application to all remaining transcripts by the lead author.

## Findings

This section presents their views of key changes to pupil wellbeing and challenges to providing effective support, including capacity in their own and external services, as well as their own wellbeing needs. It then considers staff responses to the introduction of the Framework in the light of the contextual factors presented.

### Observed Changes to Pupil Wellbeing Since the COVID-19 Pandemic

Staff at all schools noted that they had observed an increase in pupils presenting with challenges to mental health since the full-time return to in-person schooling after COVID-19:

*We've had a real increase in pupils who are suffering mental health issues, panic attacks, anxiety since coming back from Covid.* (School 7, secondary)

This was a challenge in both primary and secondary-age pupils:

*Particularly anxiety we’re noticing now, I think for a lot of our children. And you see that in their behaviour, and they just can’t control that can they?* (School 12, primary)

A range of drivers for this were discussed, including loss of social interaction leading to fears about returning to classrooms, the overall fear generated by living with the threat of the virus and, in some cases, loss of a positive adult relationship which for some, may partially mitigate a challenging home environment:

*We’ve talked about some of our students, their home life isn’t great, so to be stuck at home, we still don’t know the major impact that that’s had on them. We’re still unpicking that, things are still coming out that could be related from two or three years back.* (School 11, secondary)

At schools in areas of higher socio-economic deprivation, some staff noted that the economic challenges in those areas, exacerbated by the increased cost of living and falls in disposable income being experienced by many in the UK in recent years (Statista, 2024), were causing increased strain on families which was then likely to increase pupil mental health challenges:

*It’s that cohort here isn’t it compared to other schools, we have a lot of …yes, parents have got mental health issues as well haven’t they here which is hard. Financial issues as well, all the health issues...* (School 8, Primary)

All schools described reductions in pupil attendance since Covid, which they associated with all of these issues. This creates potential for a hidden cohort of pupils who are experiencing mental health challenges and unable to access support in the school setting.

### School-Level Barriers to Providing Support for Pupil Mental Health and Wellbeing

#### Lack of available support provision

We asked staff to consider what made it more difficult to support the mental health and wellbeing of their pupils and found significant overlap across schools, with key issues of lack of capacity in both internal and external services highly relevant.

Schools all reported existing—although highly variable—service provision in place for supporting mental and emotional wellbeing, prior to the Framework. Many staff referenced pre-existing participation in branded national programmes with a focus on mental and emotional wellbeing and social and emotional learning, as well as a range of staff training in related areas. Schools were often likely to describe themselves as ‘ACEs informed’, with practice guided by the content of a key report from Public Health Wales in 2015 on Adverse Childhood Experiences (PHW 2015) and subsequent training provided to schools.

In response to the increased issues emerging since Covid as outlined above, all schools reported increases in demand for in-house support for mental and emotional wellbeing, including increased attendance at school counselling services, as well as more staff time being spent on one-to-one support for pupils. For those pupils with more complex mental health needs, the process in schools includes referral to primary care services, specifically Child and Adolescent Mental Health Services (CAMHS). Many staff, across all areas of Wales, described a long-standing absence of capacity in these services, which had been exacerbated by the pandemic:

*They’re (CAMHS) so overstretched and overloaded, it falls back to us then doesn’t it because we’re with them day in day out.* (School 8, primary)

This created multiple challenges and concerns. While interviewees were often experienced and skilled in pastoral care, several described also being conscious of not having the training and qualifications for dealing safely with more complex cases, resulting in fear of the wrong response, as well as concern for their pupil amid the potential escalation of the issue.

#### Challenges Relating to Space and Funding

As part of their support provision, most schools we visited had a dedicated nurture space where pupils can go for anything from a brief time out to the whole day, with increased use observed at high stress times such as breaks and lunch. These were aimed at pupils who were struggling with emotional regulation and/or experiencing anxiety during the school day and ranged from whole classrooms set up as nurture spaces, to smaller corners of spaces such as the school library. Some of these spaces had been in place for several years but in many cases staff reported trying to expand them since Covid due to increased need among pupils. This was creating challenges for providing appropriate staffing and also where space in school buildings was already limited, particularly for some smaller primary schools or in sites where pupil numbers had increased in recent years.

Many staff referenced funding for extra provision as a constant challenge, with school budgets for wellbeing support not seen as growing at the rate of demand on services. In some sites, this had resulted in staff paying for supplies and equipment themselves, including one primary school who cited ongoing efforts to find additional funds from community grants, but where a staff member had self-funded the provision due to lack of other options:

*P1:* *It* (the nurture room) *is not finished, it’s nowhere near finished. You seem to have a little try at doing it and then something else happens.*

*P2:* *She’s* (the pastoral lead) *spent hundreds over the years*. (School 8, primary).

A majority of staff held favourable views of about the impact of such spaces as part of their overall provision for pupils but were highly conscious of demand outweighing supply:

*But there’s potentially quite a lot more children out there that would benefit from what we do but we just can’t cope with them all really*. (School 7, secondary).

As noted, the reported increases in pupil mental health and wellbeing challenges, coupled with lack of capacity in primary care services, means schools are holding pupils in place more frequently and, while nurture spaces appear to be a valued part of that support, there are challenges to sustainability of such provision where space and financing are limited.

#### Staff Wellbeing and Workload Pressures

At all schools, staff wellbeing was also cited as key in their ability to deliver support for pupil mental health and wellbeing, with significant increases in stress associated with maintaining school life during the pandemic and the changes in pupil needs since return to the classroom. Some discussed ongoing lack of sleep as well as impacts on their health and functioning:

*I can’t remember what happened in the last week. I’ve had phone calls with parents that have sent me a letter, thanking me for the phone call I had with them, and I genuinely can’t remember that. We all know that that’s a stress thing isn’t it?* (School 3 secondary)

While several people talked positively about the support received from their peers and school leaders, the increased needs of pupils meant that time for staff to engage in support activities for themselves and their colleagues was limited:

*It’s time, it’s time for staff to… Staff have got to give up their breaks, their lunches maybe to have a restorative conversation* (with a pupil) *and they need their break and lunch because staff wellbeing’s important.* (School 11, secondary)

While it was commonly accepted that school staff have a key role in supporting learner wellbeing, some suggested that requirements for delivery of wellbeing provision, including the Framework, ALN reforms and the new Curriculum for Wales, were actually creating additional time pressures which made support provision harder:

*You can find within lessons if you start doing wellbeing it can create such conversations, which is right, but again you haven’t got enough time [to] manage these conversations…to be honest, it’s time…there’s so much going on, and I think anxiety and well, wellbeing and mental, you know, mental health, has nearly overtaken the education…(*School 5, secondary)

Time and workload pressures were exacerbated by high levels of sickness and challenges in retention of staff, which were described as particularly challenging at the time of interviews and in a cyclical relationship, with staff absence due to workload and stress then meaning more pressure for those remaining staff, increasing the risk of additional sickness absences.

### School Staff Awareness of and Engagement with the Framework

The preceding section outlines some of the challenges occurring in schools after return from COVID-19 restrictions, both for pupils and staff. The Framework was launched during this period, with the expectation of action towards implementation at the time of data collection. This section discusses staff interactions with the Framework in light of the issues raised and any impacts on schools practice.

#### Barriers to Implementation

Levels of engagement and awareness with the Framework were highly varied, ranging from feeling familiar with content, to knowing it was there without any depth of knowledge to not having heard of it at all. Levels of engagement with the Framework did not correlate with the level of mental health and wellbeing support available for pupils at school, with a significant amount of support already in place in most settings.

Some found the Framework document itself to be challenging to engage with, with several simply arguing that it was too big and time-consuming. Others noted that the ongoing recovery from COVID-related challenges already discussed was a factor in their ability to engage with anything else:

*P2: I think with the Covid years, I not, I’m not aware, you might actually ask one of our senior management. It could have been in an inset. I can’t recall anything to do with the Framework for mental health and wellbeing?* (School 12, primary)

The demands on staff time already discussed were evident in acting as a barrier to engagement with the Framework, with other more immediate issues having to take precedent:

*…we’ve parked it at the moment because of the other things we’re dealing with.* (School 7, secondary)

As stated, the Framework was launched at a time of large-scale change to the education system, with the new Curriculum for Wales and other reforms creating competing demands on staff time. For some interviewees, a focus on implementation of the new curriculum was more significant than the Framework in shaping practice at present, which is perhaps unsurprising within educational settings.

#### Facilitators of Implementation

As part of implementation, the Welsh Government provided funding to Public Health Wales (PHW), to develop a self-evaluation tool for schools and to employ seven regional leads to raise awareness of the Framework and support schools in completion of this tool. The aim of the tool is to allow schools to assess their own practices and identify areas for improvement, in line with the Framework (see—Whole School Approach to Emotional and Mental Wellbeing—Public Health Wales (nhs.wales)).

In those schools who described the most engagement with the Framework, the role of the PHW regional leads was the strongest facilitator of this, with their role significant in making schools aware of the new guidance at a time of change and challenge in the education system, where it could have been lost to competing issues. This included speaking to wider staff teams and attending regional events:

*So she* (area lead) *leads on the framework in XXXX, so I’ve had different meetings with her, but she came along and did the framework with the staff, talked a lot about staff wellbeing and generated a lot of discussion*. (School 11, secondary)

While schools are not required to engage with this self-evaluation tool in order to implement the Framework, it is recommended by Welsh Government as a first step and, for many, the actual Framework and the self-evaluation tool promoted by area leads tended to be seen as one and the same, often referred to interchangeably, illustrating the importance of this direct in-person implementation support. For those who had commenced or completed the self-evaluation, it was described as a helpful means of consolidation of existing practice and identifying new priorities. It was generally agreed that the actions outlined in the Framework were a good ‘fit’ with existing ethos and wellbeing practices in these schools, rather than representing a radical departure from current approaches:

*I think the majority of the teachers, if not all of them, have got the kids’ wellbeing as a priority rather than anything else. I know we’re all gunning for results, but you know when those kids are having an off day.* (School 4, secondary)

In line with the challenges on staff time already highlighted, this was again significant in engagement with the Framework. Where schools were able to release staff from other duties or use staff inset days to discuss and work on the self-evaluation tool (with the attendance of PHW area leads at these inset days), this was seen as highly supportive:

*We arrange for people to sign up for whichever day they wanted a supply in and then the supply comes in and does a wellbeing day for that class whilst the teacher is out doing the self-assessment.* (School 8, primary)

However this was also acknowledged as challenging, often with funding required to provide cover for staff released for this work, making it prohibitive for some schools. It was noted, including by some in those schools who had already invested time and money, that for new statutory requirements to be implemented comprehensively and sustainably, ongoing financial support, specifically from the Welsh Government would be key:

*…they have to realise that they are going to take resources and they need to fund it, the Welsh Government. It’s all very well putting it in the budget, in the curriculum, but I think they really, and the only way to, in my opinion, are resources for people as well…* (School 12, primary).

## Discussion

This research explored contextual factors within a sample of schools at the time of the launch of the Whole School Approach Framework in Wales from the perspective of school staff. It aimed to consider barriers and facilitators to changes in school practice in relation to mental wellbeing and stakeholders responses to initial Framework implementation.

Findings illustrate the importance of considering both how the internal complexity evident in school systems impacts implementation but also the key role of the bigger picture in which change occurs (Egan et al., 2019). Data showed that this was a time of significant pressures on the school system in Wales, with COVID-19 still highly disruptive to school function due to an observed growth in the scale and complexity of pupil wellbeing problems, as well as ongoing impacts on staff wellbeing and retention. In the wider context around schools, external pressures were also evident, including lack of capacity in specialist support services meaning pressure on school staff to deal with more complex mental health challenges internally.

Staff at all schools described an increase in pupil mental health challenges since COVID-19, with expressed concerns over their own capacity to respond to this and the strain on existing school wellbeing provision. As highlighted by interviewees, interaction of the setting with local and national context was also important in implementation (Skivington et al., 2021). Current UK economic challenges were adding to issues stemming from COVID-19, amplified in areas with higher levels of deprivation pre-pandemic, supporting evidence on the impact of the local socio-economic context of the school and family on pupil mental health (Dalmaijer, et al., 2023; Higgins & Booker, 2022). This adds to the strain on school services and risks escalation of emerging issues without support for schools in increasing their capacity, for example in school nurture spaces and counselling services. Further, the pre-existing absence of capacity in specialist services was an ongoing problem in light of the increased demand noted here, with challenges likely to be exacerbated by existing inequalities in access for those in more deprived areas (Fairchild, 2019). The lack of specialist care has implications for delivery of the desired Framework outcomes of improving mental health and better links between schools and external agencies, illustrating the importance of considering the wider system when implementing new programmes. For these outcomes to be met, as well as to reducing inequalities in mental health challenges, policy-makers should consider investment in specialist services to increase capacity (Khanal et al., 2021).

School staff are the key delivery agents for the Framework, and data suggest that there ability to deliver system-wide change was hampered by multiple challenges. Interviewees were highly likely to state that their own wellbeing and that of their colleagues was poor, impacting their capacity to support pupils. Increasing expectations over recent years for school to act as, not only educators, but as key providers of wellbeing and developmental support (Long et al., 2023) risks adding to increasing pressure on staff who may feel that they lack the relevant skills or training for this. Effective implementation of school programmes requires both the ‘will and the skill’ from school staff (Lendrum et al., 2013), including feeling both prepared and equipped for new duties (Fallon et al., 2018). Policy-makers should consider staff wellbeing, not only as an end product of Framework implementation, but as a key mechanism of delivery. The Framework was largely viewed as a good fit with existing systems rather than something displacing current approaches (Hawe et al., 2009). However, although the ethos was complementary to existing work on mental health, implementation was reported as burdensome where staff were already feeling time poor and highly stressed. Evidence suggests that teachers respond best to wellbeing initiatives that are whole school and include focus on reducing burden and increasing feelings of competence (Brady & Wilson, 2021). A wellbeing programme that requires significant time and effort from school staff is likely to be counterproductive where pre-existing system pressures are not considered. Further, the range of initiatives impacting the Welsh education landscape may be detrimental to their wellbeing, with fears over keeping up with the pace of change (Glazzard & Rose, 2020) and the risk of cynicism and overload in a constantly changing landscape of new initiatives (Lendrum et al., 2013). Where staff wellbeing is already poor, even where the aim is long-term improvement in this, there is a potential risk of implementation failure due to those key delivery agents simply being overwhelmed.

Policy-makers should apply a complex-systems lens to consider the range of personal and organisational factors that here impacted not only initial responses to the Framework, but likely ongoing implementation. These include ensuring staff support for the programme which may be increased by use of inset days for training (Moir, 2018). Consideration should also be given to support for school leadership in increasing staff knowledge and beliefs about the programme (Hudson et al., 2020). Here, knowledge of the Framework was highly variable among interviewees and, at the time of interview, the Framework did not appear to be cascading through staff teams on a consistent basis. The Framework contains recommendations for action (through self-evaluation) and a directive that schools must attend to the content, but with no mandated timeline, implementation guidance or performance indicators, which may have contributed to the high degree of variation in school awareness and engagement with the programme. For those who had initiated implementation and acted on the recommendation to self-evaluate, this was strongly driven by the work of the PHW area leads and the support provided, highlighting the importance of implementation support for organisational change (Fallon et al., 2018). As highlighted by participants, for the Framework to become embedded in school systems, this is likely to require sustained resourcing after initial self-evaluation. Further, it should be acknowledged that change resulting from the Framework is more likely to be consolidatory than transformative, as schools were already providing a wide range of support for mental and emotional wellbeing.

## Limitations

While case study schools were selected to capture a diverse range of experiences of implementation, it is possible that other school staff would report different approaches and interpretations. However learning from these findings is likely to have some transferability, such as the increased demand since return from lockdowns during the COVID-19 pandemic, challenges to staff time, workload and wellbeing. These are likely to continue to present significant barriers to programme sustainability.

Participants were selected due to their involvement in pupil wellbeing support; however, where those staff had little or no knowledge of the Framework, it cannot be guaranteed that no-one in that particular school did not have that knowledge. It does however imply that the Framework had not yet disseminated widely among the staff team and gained any traction in day-to-day working.

## Conclusion

Study findings suggest key considerations for policy-makers involved in the delivery of a whole school approach to emotional and mental wellbeing. While findings were generated in a Welsh context, they are likely to have transferability to other settings, including other UK nations, where whole school approaches are being introduced or considered. Firstly, consideration should be given to how to improve capacity in both in-house mental wellbeing services within schools as well as community-based services. This is essential due to the increased demand for support noted since COVID-19, as well as the policy demand for schools to be more responsive to mental health challenges. Secondary, support and funding for staff training is needed to ensure that staff feel confident in encouraging pupil disclosures and responding appropriately. This should also include clear guidance for school senior leaders on how to support implementation of the Framework in their schools. Thirdly, consideration should be given to increasing the provisions of staff wellbeing services, both as an end in itself and as a valuable aid in supporting staff to feel more able to support their pupils. Finally, long-term support for implementation is key to understanding how the Framework changes schools, with evidence that it can take 3–4 years for evidence led programmes to become embedded in systems and significantly longer for observable outcomes (Ogden et al., 2012).
